# Plastome Sequence Determination and Comparative Analysis for Members of the *Lolium*-*Festuca* Grass Species Complex

**DOI:** 10.1534/g3.112.005264

**Published:** 2013-04-01

**Authors:** Melanie L. Hand, German C. Spangenberg, John W. Forster, Noel O. I. Cogan

**Affiliations:** * Department of Primary Industries, Biosciences Research Division, AgriBio, the Centre for AgriBioscience, La Trobe University Research and Development Park, Bundoora, Victoria 3083, Australia; † Dairy Futures Cooperative Research Centre, Bundoora, Victoria 3083, Australia; ‡ La Trobe University, Bundoora, Victoria 3086, Australia

**Keywords:** Italian ryegrass, meadow fescue, tall fescue, perennial ryegrass, chloroplast DNA, phylogenetics

## Abstract

Chloroplast genome sequences are of broad significance in plant biology, due to frequent use in molecular phylogenetics, comparative genomics, population genetics, and genetic modification studies. The present study used a second-generation sequencing approach to determine and assemble the plastid genomes (plastomes) of four representatives from the agriculturally important *Lolium-Festuca* species complex of pasture grasses (*Lolium multiflorum*, *Festuca pratensis*, *Festuca altissima*, and *Festuca ovina*). Total cellular DNA was extracted from either roots or leaves, was sequenced, and the output was filtered for plastome-related reads. A comparison between sources revealed fewer plastome-related reads from root-derived template but an increase in incidental bacterium-derived sequences. Plastome assembly and annotation indicated high levels of sequence identity and a conserved organization and gene content between species. However, frequent deletions within the *F. ovina* plastome appeared to contribute to a smaller plastid genome size. Comparative analysis with complete plastome sequences from other members of the Poaceae confirmed conservation of most grass-specific features. Detailed analysis of the *rbcL*–*psaI* intergenic region, however, revealed a “hot-spot” of variation characterized by independent deletion events. The evolutionary implications of this observation are discussed. The complete plastome sequences are anticipated to provide the basis for potential organelle-specific genetic modification of pasture grasses.

Sequences of plant plastid genomes (plastomes) have been long used for a broad range of plant biology applications. Due to the properties of high sequence conservation and abundance (such that thousands of copies may be present in each cell) (Zoschke *et al.* 2007) plastomes, or regions thereof, have been used for phylogenetic studies (Burke *et al.* 2012; Wu and Ge 2012; Zhang *et al.* 2011b), comparative genomics (Gao *et al.* 2010), DNA barcoding activities (Hollingsworth *et al.* 2009), and various biotechnology applications (Clarke *et al.* 2011). The plastome is a circular molecule that, in most taxa, varies between 108 and 218 kb in size and generally displays a high level of conservation between land plant species. The typical plastome is composed of two identical inverted repeats (IR, 20–76 kb) that separate a large single-copy (LSC, 60–90 kb) and a small single-copy (SSC, 7–27 kb) region (Chumley *et al.* 2006). Generation of a complete plastome sequence from representative species can hence provide information related to evolutionary history, and permit the development of sequence-based tools such as molecular genetic marker assays for ecological studies and DNA barcode-based diagnostics for species identification. Traditionally, plastome sequencing and assembly has involved the extraction of high-purity chloroplast DNA, which is then cloned into vectors and sequenced using dideoxynucleotide terminator chemistry. However, as second-generation technology-based sequencing projects have become more cost-effective and feasible, a number of recent studies have exploited these methods to assemble plastome sequences from total cellular DNA templates. Target species for this approach have included rice (*Oryza* spp.) (Nock *et al.* 2011), pear (*Pyrus pyrifolia* [Burm.] Nak.) (Terakami *et al.* 2012), duckweed (Lemnoideae spp.) (Wang and Messing 2011), and date palm (*Phoenix dactylifera* L.) (Yang *et al.* 2010).

Grasses of the genera *Lolium* and *Festuca* (Poaceae family, Pooideae subfamily) include a number of agriculturally important pasture grasses that are widely cultivated in temperate regions, including perennial ryegrass (*Lolium perenne* L.), Italian ryegrass (*L. multiflorum* Lam.), meadow fescue (*Festuca pratensis* Huds. syn. *L. pratense* Huds. [Darbysh.]), and tall fescue (*F. arundinacea* Schreb. syn. *L. arundinaceum* Schreb. [Darbysh.]). In addition, the *Festuca* genus contains a number of grass species grown for turf or ornamental purposes such as sheep’s fescue (*F. ovina* L.) and red fescue (*F. rubra* L.). The *Festuca* genus is recognized as containing more than 600 species with multiple ploidy levels and near-global distribution, whereas the *Lolium* genus contains only 10 recognized diploid taxa (Clayton and Renvoize 1986). The two genera have been differentiated on the basis of inflorescence structure, but controversy has surrounded the taxonomic classification of some *Lolium* and *Festuca* species. Phylogenetic analysis consistently identifies the *Lolium* genus as being nested within the *Schedonorus* subgenus of the *Festuca* genus (Catalán *et al.* 2004; Charmet *et al.* 1997; Hand *et al.* 2010; Inda *et al.* 2008; Torrecilla and Catalán 2002; Xu and Sleper 1994), and the species complex has been subject to reclassification (Darbyshire 1993; Soreng and Terrell 1997).

Regardless of the taxonomic classification system that is used, it is clear that the *Lolium* and *Festuca* genera represent a closely allied complex of related and partially interfertile species. Further complexity is present within the allohexaploid species tall fescue, which may also be more accurately described as a species complex, as three eco-geographic races (morphotypes) are recognized (Hand *et al.* 2010), at least two of which (Continental and Mediterranean) differ in terms of ancestral genome origin. Of the three, the Continental morphotype has been the most widely cultivated and studied at the genetic level. Within the *Lolium-Festuca* species complex, complete plastome sequences have so far been assembled from perennial ryegrass and hexaploid tall fescue (the Continental cultivar KY31) (Cahoon *et al.* 2010; Diekmann *et al.* 2009).

Complete plastome sequences from members of the *Lolium-Festuca* species complex would be valuable for further study of these taxa and for a broader understanding of Poaceae plastome evolution. Since publication of the first plastome sequence from a grass species (Hiratsuka *et al.* 1989), subsequent comparisons with that of tobacco (*Nicotiana tabacum* L.), which is regarded as the reference for angiosperms, has revealed six main structural alterations. These changes include three inversions in the LSC region, loss of an intron in the *rpoC1* gene, a sequence insertion in the *rpoC2* gene (Shimada *et al.* 1990), rearrangement of the *accD* gene (Ogihara *et al.* 1992), absence of ORFs within the IR (Downie *et al.* 1994), and translocation of the *rpl23* gene to a region of the LSC region, between *rbcL* and *psaI* (Ogihara *et al.* 1988). As more grass plastome sequences became available, these structural alterations have been studied further and the timing of their origin has been discussed (Harris *et al.* 2013; Katayama and Ogihara 1996; Morris and Duvall 2010). However, a definitive understanding of variation present within Poaceae plastomes is yet to be obtained.

This study describes the assembly of complete plastomes from a selection of four representative species within the *Lolium−Festuca* species complex, using sequence generated from total cellular DNA based on second-generation technology. The resulting plastomes were compared with those of other Poaceae species to compare higher-level organization and gene content and to support phylogenomic analysis of evolutionary divergence within the *Lolium-Festuca* species complex.

## Materials and Methods

### Plant material and DNA extraction

Sampling of diploid *Lolium-Festuca* species was designed to include important agricultural species, along with representatives of each major subgenus within the *Festuca* genus (Inda *et al.* 2008; Torrecilla and Catalán 2002). Seed was sourced from either Heritage Seeds Australia, Seed Force Australia, the USDA germplasm collection, or the Genetic Resources Unit of the Institute for Biological, Environmental and Rural Studies (Aberystwyth, Wales). Details of the four selected accessions are provided in Table 1. This study represents an extension of a pre-existing shotgun whole-genome sequencing activity for each of the included diploid species, which aimed to enrich the proportion of sampled nuclear genome through selection of target tissue for DNA extraction. For meadow fescue and Italian ryegrass, DNA was consequently extracted from root tissue of plants grown in sand pots, and roots were washed with distilled water and then ground in liquid nitrogen prior to extraction. As root tissue was unavailable from the *F. altissima* and *F. ovina* accessions due to quarantine restrictions, DNA was extracted from leaf tissue for these samples. All DNA extractions were performed using the DNeasy Plant Mini Kit (QIAGEN, Hilden, Germany).

**Table 1:** Details of the four species selected for plastome sequencing

Species	*Festuca* Subgenus	Source	Cultivar/Accession	Plant Tissue
*Lolium multiflorum*	−	Seed Force	Accelerate	Root
*Festuca pratensis*	Schedonorus	USDA germplasm collection	Mimer (PI 310482)	Root
*Festuca altissima*	Drymanthele	IBERS	BS4384	Leaf
*Festuca ovina*	Festuca	IBERS	BL2643	Leaf

USDA, United States Department of Agriculture; IBERS, Institute for Biological, Environmental and Rural Studies.

### Paired-end library preparation and sequencing

For each sample, paired-end libraries with approximately 200-bp inserts were prepared using the Illumina TruSeq DNA Sample Preparation Kit (Illumina Inc., San Diego, CA). Library quantification was performed using the KAPA Library Quantification Kit (KAPA Biosystems, Boston, MA). Paired-end cluster generation and sequencing was performed on the Hi-Sequation 2000 (Illumina), with each library being allocated to one lane of a flow cell. All generated sequence reads were subjected to quality control using a custom PERL script and were trimmed if they met any of the following criteria: more than three consecutive nucleotides designated as unattributable base identities (N); more than three nucleotides had a PHRED quality score less than or equal to 20; or a median PHRED quality score less than 20. Furthermore, reads were discarded when the length was less than 25 nucleotides.

### Estimation of bacterial and plastid genome contribution

For each sequenced genotype, the proportions of sequence reads of probable bacterial or plastid genome origin were estimated using a reference alignment approach. A subset of reads from each genotype was mapped to a set of generated reference sequences consisting of complete or partial genomes for 43 bacterial species and five chloroplast genomes (Supporting Information, Table S1). Alignment was performed using the Burrows-Wheeler Alignment (BWA) tool (Li and Durbin 2009) with both the maximum number of gap openings and edit distance in the seed set as five. All other parameters were left as default values. File format conversion was performed using the utilities for the Sequence Alignment/Map (*i.e.*, SAM) format (SAMtools) (Li *et al.* 2009), and the alignment was ultimately viewed with Tablet 1.12.02.06 (Milne *et al.* 2010).

### Plastome assembly

Sequence reads of plastome origin initially were filtered from the sequencing output by alignment of all reads to the published chloroplast genomes of perennial ryegrass, tall fescue, bread wheat (*Triticum aestivum* L.), barley (*Hordeum vulgare* L.), and *Brachypodium distachyon* L. (GenBank accession numbers NC009950, FJ466687, NC002762, EF115541, and EU325680). Alignment was performed using BWA, as described previously.

For each species, all reads that aligned to any of the chloroplast genome references were extracted and *de novo* assembled using SOAPdenovo v1.05 (127mer version; (http://soap.genomics.org.cn) (Li *et al.* 2010). A range of k-mer values were tested (ranging from 51 to 91), and the final assembly was selected when the contigs had an additive length similar to that of the perennial ryegrass chloroplast genome (135,282 bp). Contigs generated in the SOAPdenovo assembly were further assembled in Sequencher 4.7 (GeneCodes Corp., Ann Arbor, MI) with a specification of minimum match percentage of 97% and a minimum overlap of 50 bp. Contigs were ordered on the basis of the pre-existing annotation of the perennial ryegrass chloroplast genome. Gaps between contigs were presumed to derive from plastome regions that are sufficiently divergent between the reference genomes and the sequenced species, such that no reads mapped to these regions, hence preventing inclusion in the *de novo* assembly. The initial attempt to fill gaps therefore exploited the sampling strategy and iteratively used contigs of the next most closely related species as a reference for filtering sequence reads of plastome-origin via BWA mapping. Any gaps remaining after completion of this process were subsequently amplified by polymerase chain reaction (PCR) using primers designed to flanking regions and sequenced using dideoxynucleotide terminator chemistry. PCR conditions were as previously described (Hand *et al.* 2010), and cycling conditions included an enzyme activation step of 95° for 15 min, followed by 30 cycles of 95° for 1 min, 65° for 30 sec, 72° for 1 min with the annealing temperature decreasing for 1° per cycle until the final temperature was 55°, and a final extension step of 72° for 10 min. PCR products were purified and directly sequenced, as described previously (Hand *et al.* 2010).

As the plastome contains two identical copies of an inverted repeat (IRa and IRb), the assembly was initially performed to generate a contig that included only the LSC, IRb and SSC sections. The IRa section was subsequently added manually to the contig, and IR boundaries were confirmed using PCR.

### Genome annotation, alignment, and phylogenomic analysis

Each complete plastome was annotated using the online software DOGMA with default parameters (Wyman *et al.* 2004). Predicted coding regions were visually inspected, compared with the published chloroplast genomes of perennial ryegrass and tall fescue, and adjusted accordingly. Complete plastome sequences of 12 species were aligned using the LAGAN program within the mVISTA online suite of computational tools (Brudno *et al.* 2003). Default parameters were applied, and the annotation framework of the perennial ryegrass chloroplast genome was used. Percentage identity between each plastome, all relative to that of perennial ryegrass, was subsequently visualized through a VISTA plot (Frazer *et al.* 2004).

Plastome-based phylogeny was reconstructed for the six *Lolium-Festuca* species using whole plastome alignment generated by LAGAN, as described previously. Plastomes of the Pooideae species creeping bentgrass (*Agrostis stolonifera* L.) and barley (GenBank accession numbers NC008591 and EF115541) were also included as outgroups. The phylogenetic tree was constructed through the method of maximum parsimony as implemented by MEGA 5.10 (Tamura *et al.* 2011). Sites with gaps or missing data were excluded from the analysis, and statistical support was achieved through bootstrapping using 1000 replicates.

### Analysis of grass-specific plastome features

Structural features of the plastome that had previously been identified as grass-specific were examined for 12 Poaceae species, including each of the four species selected for this study. The structure of *rpoC2* was analyzed by aligning this gene from each species using Sequencher 4.7 (GeneCodes), and the insertion event within this gene was detected using previously defined sequence boundaries (Katayama and Ogihara 1996). The *rpl32′* pseudogene was deemed to be present within a plastome if the *rpl32* gene predicted by DOGMA was located between *rbcL* and *psaI*. Similarly, identification of an *accD* pseudogene was determined based on prediction of an incomplete form by DOGMA. To further investigate the extent of inter-specific variation, the intergenic region between *rbcL* and *psaI* of the 12 Poaceae species, was aligned using Sequencher 4.7 (GeneCodes) and manually edited as required. Only deletions greater than 40 bp were recorded and represented within the diagram.

## Results

### Sequencing output

**Figure 1:**
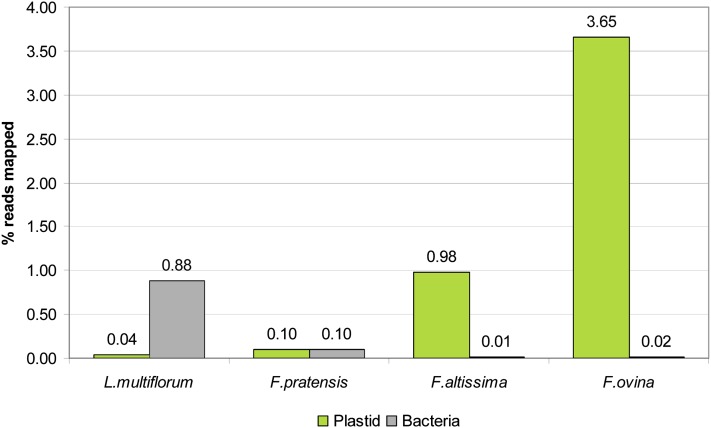
Proportion of sequence reads identified as originating from either plastid or bacterial genomes, based on sequencing output from each species. The percentages of reads mapped to each of the reference genome sets are displayed above each bar.

Variation was observed between samples with respect to the proportion of reads predicted to originate from either plastid or bacterial genomes (Figure 1). For instance, the library constructed from Italian ryegrass root tissue contained substantially more bacterium-derived DNA than libraries constructed for any of the other species. The bacterial reference genome which was predominantly responsible for this large discrepancy belonged to the species *Flavobacterium johnsoniae*. A direct comparison of the root- and leaf-extracted DNA templates revealed that the former contained fewer (average of 26-fold) plastome reads, but a greater number of reads from bacterial genomes (average of 28-fold).

### Plastome assembly and organization

Complete plastomes for each of the included species were successfully assembled following sequencing of total DNA templates and submitted to GenBank under the accession numbers JX871939-JX871942. As expected, the read filtering process recovered a greater number of plastid genome reads from the leaf-extracted DNA template (3.6 × 10^6^ and 7.9 × 10^6^ reads from *F. altissima* and *F. ovina*, respectively) than the root-extracted DNA template (1.1 × 10^5^ and 4.7 × 10^5^ reads from Italian ryegrass and meadow fescue respectively). Throughout the assembly process, a larger number of unclosed gaps were present for *F. altissima* and *F. ovina*, as compared to plastomes of the other two species. However, all gaps present within the initial assembly were capable of closure using PCR.

The complete plastomes of the four sequenced species ranged in size from 133,165 bp to 135,291 bp, similar to those of perennial ryegrass and tall fescue (Table 2). For each plastome, the LSC and SSC regions were c. 80 kb and 12 kb in size, respectively. The LSC and SSC regions were separated by the pair of inverted repeats (IRa and IRb), which were both c. 21 kb long (Table 2).

**Table 2:** A comparison of plastome size and organization for six *Lolium-Festuca* species

					% of Plastome Identified as:		
	Plastome Size	LSC Size	SSC Size	IR Size	Gene Space	Intron Space	Intergenic Space
*L. perenne*	135,282	79,972	12,428	21,441	54.34	11.91	33.75
*L. multiflorum*	135,175	79,848	12,485	21,421	53.56	12.01	33.49
*F. pratensis*	135,291	79,934	12,511	21,423	53.54	11.91	33.61
*F. arundinacea*	136,048	80,560	11,300	22,600	54.20	9.31	36.49
*F. altissima*	135,272	79,828	12,598	21,423	53.64	11.99	33.44
*F. ovina*	133,165	78,329	12,386	21,225	54.50	12.19	32.33

All genome sizes are denoted in base pairs.

The annotation process identified 114 different genes within the plastomes of the sequenced species. Of these, 20 were duplicated within the IR regions, which brought the total to 134 genes. A total of 82 genes were present within the LSC and 12 within the SSC region. The gene space accounted for approximately 54% of each genome, the remaining sequence being attributed to intergenic spacers (c. 33%) and introns (c. 12%) (Table 2). These proportions are similar to those previously identified in the perennial ryegrass chloroplast genome, although the tall fescue plastome appears to contain a larger (36.49%) amount of intergenic space and a lower proportion (9.31%) of introns.

Annotation details for the four species in this study differ slightly from those of the published perennial ryegrass and tall fescue plastomes. In comparison, the perennial ryegrass chloroplast genome lacks three tRNA genes, and the hypothetical reading frame *ycf68* (which is located within the intron of *trn*I-GAU), although present, was not annotated. Furthermore, the tall fescue plastome lacks the *rps14* gene, *ycf4* and two tRNA genes. Of the 77 different protein-coding genes identified within the four sequenced species, 16 display length polymorphism based on comparison between the six species (Table 3). The most notable of these are *rpoC2*, *rps18*, and *ycf68*, which differ in length by 38, 21, and 18 codons, respectively. For all six species that were compared, the IR region had expanded to include a portion of the *ndhH* gene, as observed in certain other grasses. The size of this gene portion is also variable among the six *Lolium-Festuca* species, varying from 60 to 67 codons in length.

**Table 3:** List of plastome genes that vary in size between the six *Lolium-Festuca* species included in the comparative study

	Gene Size in Codons						
Gene Name	*L. perenne*	*L. multiflorum*	*F. pratensis*	*F. arundinacea*	*F. altissima*	*F. ovina*	Size Difference
*atpA*	508	508	508	508	508	505	3
*atpI*	248	248	248	248	248	247	1
*cemA*	231	231	231	231	231	226	5
*clpI*	217	217	217	215	217	215	2
*infA*	108	108	108	114	114	114	6
*ndhD*	503	503	503	501	501	501	2
*ndhF*	742	742	742	739	740	740	3
*ndhH**^a^*	60	60	60	60	63	67	7
*psbF*	40	40	40	40	43	40	3
*rbcL*	478	478	478	488	478	480	10
*rpl32*	60	60	60	68	60	64	8
*rpoC2*	1467	1467	1474	1505	1474	1474	38
*rps15*	91	91	91	91	93	91	2
*rps16*	90	93	90	89	86	86	7
*rps18*	157	157	157	150	157	171	21
*ycf68*	−	127	127	127	145	145	18

The length of each gene is given in codons, along with the size difference between the shortest and longest variant for each gene.

a This *ndhH* gene fragment is present within the IRb region.

### Whole plastome comparison

**Figure 2:**
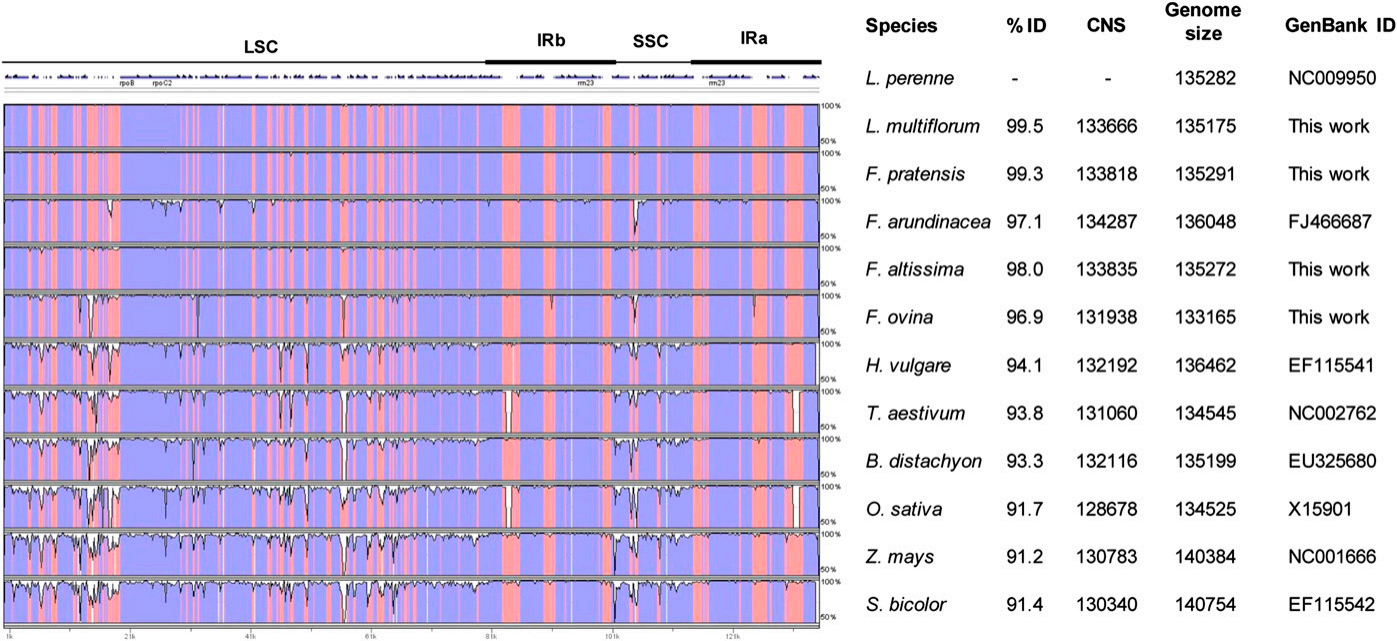
Alignment of complete plastome sequences from 12 Poaceae species. Alignment and comparison was performed using mVISTA, and percentage identity between the plastomes was visualized in the form of a VISTA plot. Each measure of similarity (% ID) is relative to the plastome of perennial ryegrass, which was used as a reference. Blue-shaded regions indicate coding regions, as defined by annotation of the perennial ryegrass. Pink regions represent conserved noncoding sequence. Regions of the plastome are illustrated above the VISTA plot. CNS, conserved nucleotide sequence.

Genome-wide comparison of the *Lolium-Festuca* plastome sequences reveals a high level of conservation (96.9%–99.5%) as visualized by the VISTA plot (Figure 2). Interestingly, the plastid genome of *F. altissima* showed greater sequence similarity to that of perennial ryegrass (98.0%) than the previously published plastome of tall fescue (97.1%). In general, the IRs displayed lower levels of sequence divergence than the single-copy regions. Divergence was most apparent within intergenic regions, closer analysis of which revealed frequent deletions within the *F. ovina* plastome, ranging between 16 and 503 bp in length. These small, intergenic deletions presumably account for the smaller genome size observed for this species.

Obvious “hot-spots” of variation occur between the *psbC* and *rpoB* genes, the intergenic regions between *rps4* and *clpP*, and a small area within the SSC region, between the *ndhF* and *ccsA* genes. The similarity of plastome organization that the six species share with other members of the Poaceae family is also apparent from the VISTA plot. Within the broader Poaceae, the genes also appear to be well conserved, and the major sites of divergence are located within the aforementioned regions. Compared with other Poaceae species, the wheat and rice plastomes possess an IR-located deletion between the *rpl23* and *ndhB* genes of c. 1 kb, which is likely to partially contribute to smaller observed plastome sizes.

### Grass-specific plastome features

Each plastome generated in this study was examined for the presence of previously identified structural features that are unique to grasses and subsequently was compared with other Poaceae plastomes. As with most other Poaceae species sequenced to date, each plastome contained three previously defined inversions within the LSC and failed to contain an intron within the *rpoC1* gene. *Lolium* and *Festuca* plastomes also displayed an insertion within the *rpoC2* gene compared with the reference dicotyledonous plant plastome sequence (of *N. tabacum*). The size of this insertion varies within the Poaceae and ranges from 341 to 438 bp in length (Table 4).

**Table 4:** Details of the variable grass-specific plastome features for 12 Poaceae species

	*rpoC2* Insertion Size, bp	*rpl32′* Presence	*accD* Presence	*rbcL-psaI* Region, bp
*L. perenne*	341	Absent	Pseudo	1183
*L. multiflorum*	341	Absent	Pseudo	1182
*F. pratensis*	362	Absent	Pseudo	1205
*F. arundinacea*	419	Absent	Pseudo	1206
*F. altissima*	362	Absent	Pseudo	1179
*F. ovina*	362	Absent	Absent	885
*H. vulgare*	407	Present	Pseudo	1603
*T. aestivum*	413	Present	Absent	879
*B. distachyon*	383	Absent	Absent	461
*O. sativa*	386	Present	Pseudo	1693
*Z. mays*	438	Present*^a^*	Absent	888
*S. bicolor*	417	Present	Absent	861

a Detected in this study although not annotated in GenBank.

Variation also was detected in the presence of an *rpl23* translocation product (*rpl23′*) and an *accD* pseudogene in the region between *rbcL* and *psaI* (Table 4). The *rpl23′* element was absent from the plastomes of all *Lolium-Festuca* species, as well as *B. distachyon*. The presence of this pseudogene, however, was confirmed in all other analyzed species. Remnants of the *accD* gene were detected in almost all *Lolium-Festuca* species, with the exception of *F. ovina*. This pseudogene was also identified in barley and rice but was not predicted in the other species.

**Figure 3:**
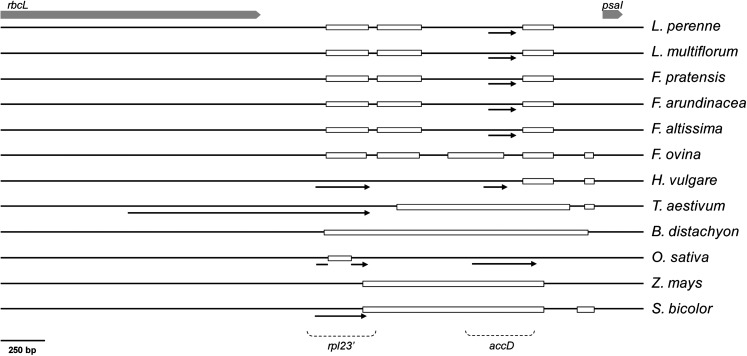
Major deletions within the *rbcL–psaI* intergenic region across 12 Poaceae species. Annotated genes from each species are depicted by a black arrow, and gene identity is illustrated at the bottom of the diagram. Deletions greater than 40 bp in length are represented by a rectangle. The boundaries of the intergenic region are defined at the top of the figure by shaded block arrows representing *rbcL* and *psaI* genes.

Alignment of the *rbcL*–*psaI* region from each Poaceae taxon revealed that differences in annotation result from deletions of varying size (Figure 3). All *Lolium-Festuca* species included in the alignment possess two deletions (of c. 250 bp each) in the position occupied by the annotated *rpl23′* element in other species. The only remaining evidence for presence of *rpl23*′ within these species is a c. 55 bp region that precedes the two deletions. The *accD* pseudogene is absent from *F. ovina* due to a 317-bp deletion which is not present in the other *Lolium-Festuca* species. Of the 12 candidate species, barley and rice possess the smallest number of deletions, and contain evidence for both the *rpl23′* and *accD* pseudogenes. Plastomes of the remaining species contain large (1–1.5 kb) deletions which have removed either one (wheat, maize and sorghum) or both of the pseudogenes (*B. distachyon*) in this region. This alignment also revealed frequent single base indels within the tall fescue plastome sequence, as compared to the other grass species.

### Phylogenomic analysis

**Figure 4:**
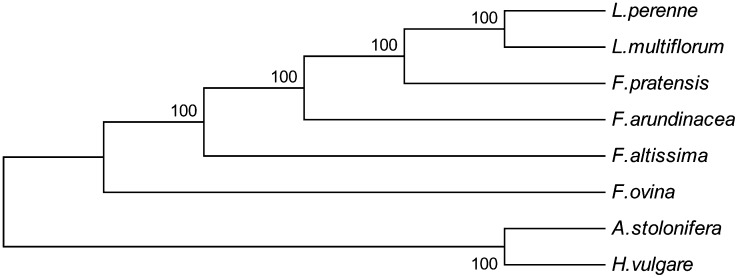
Phylogeny of *Lolium-Festuca* species based on whole plastome sequence. Plastomes of *Agrostis stolonifera* and *Hordeum vulgare* were included as outgroup species. The phylogenetic tree was drawn using maximum parsimony and bootstrap support was achieved using 1000 replicates.

Phylogenomic analysis of representatives from the *Lolium-Festuca* species complex produced a single, well-supported tree using maximum parsimony (Figure 4). The tree is fully resolved with the two *Lolium* species sister to each other and paraphyletic with *Festuca*. The two outgroup species (*A. stolonifera* and *H. vulgare*) are basal to the remaining species in a separate, resolved clade.

## Discussion

### Optimization of plastome sequencing methodology

Through extraction and sequencing of total DNA from a given sample, the genomes from each organelle (nucleus, plastid and mitochondria) are captured, along with any microbial genomes that may have been inadvertently coextracted. In the present study, generation of total DNA template from both root and leaf tissue permitted comparison of proportions of sequence reads generated from the various genomes for each source. Substantial variation was observed between species with respect to the proportions of bacterial and plastome reads. Nonetheless, a greater proportion of plastome-derived reads, as expected, was obtained from the leaf tissue-derived template, presumably due to greater abundance of chloroplasts within leaf cells. The number of plastome copies per leaf cell depends upon age and physiological state, but a ratio of more than 10,000 has been reported (Boffey and Leech 1982).

In addition to generating a smaller proportion of plastome-related reads, sequencing of the root-extracted template also suffered the disadvantage of producing a greater number of reads of bacterial origin. Possible sources for the contamination include rhizobacteria growing in close association with the root system, or bacterial pathogens present within the plant tissue (Egli *et al.* 1975; Franche *et al.* 2009; Okon and Labandera-Gonzalez 1994; Vauterin *et al.* 1995). In addition, a large proportion of the bacterial contamination possibly occurred through the inability to remove all soil particles attached to the root tissue prior to DNA extraction. *Flavobacterium johnsoniae*, which was predominantly responsible for the higher levels of contamination associated with the Italian ryegrass library, is commonly found in soil and freshwater (McBride *et al.* 2009; Stanier 1947), consistent with the assumption of soil-borne contamination. This result has broader implications for the design of whole-genome sequencing projects. The use of root tissue template for DNA extraction would be beneficial for such enterprises to maximize yield of sequence reads generated from the nuclear genome. The success of this approach, however, is obviously dependent upon extensive cleaning of source tissue to prevent inadvertent sequencing of microbial genomes.

Although presenting its own unique challenges, plastome assembly from reads representing total DNA is now undoubtedly a more rapid and efficient strategy than the previous method based on organelle fractionation.Filtering of plastome-derived reads from the remaining pool is an essential first step, which has been achieved for most studies to date through alignment of all reads to a reference plastid genome (Wang and Messing 2011; Yang *et al.* 2010; Zhang *et al.* 2011a). The success of this filtration step hence depends upon the evolutionary distance between the reference and target species, such that a more closely related reference genome would be able to recover a greater proportion of reads. This prediction was accurate for the present study, as a larger number of gaps were apparent within the plastome assemblies for those species (*F. altissima* and *F. ovina*) that are more distantly related to the perennial ryegrass reference than more closely related species (Italian ryegrass and meadow fescue). Once plastome reads have been filtered, the *de novo* assembly process is complicated by the presence of the IR regions, which are unable to be independently assembled due to sequence identity. PCR amplification was consequently required to verify the presence and precise boundaries of the IRs. Hence, although shallow sequencing of total DNA template provides a rapid and cost-effective approach for plastome assembly, PCR and first-generation sequencing are still necessary to close gaps within the assembly and, at the very least, provide a level of quality control.

### Comparison of *Lolium-Festuca* plastomes

Comparative analysis revealed that the plastomes generated in this study are highly similar in terms of organization and sequence identity, with major differences being attributable to divergence within intergenic regions. In particular, the *F. ovina* plastome contains a larger number of deletions within the intergenic regions, in comparison with those of the other *Lolium-Festuca* species. This degree of differentiation correlates with its taxonomic position because *F. ovina* is the only fine-leaved *Festuca* species to be selected in this study. Another variable region between the plastomes of the targeted species is within the IR boundary. The IR region has expanded to include a portion of the *ndhH* gene, in common with other species of the Pooideae subfamily (Ogihara *et al.* 2000; Saski *et al.* 2007). The extent of this expansion, however, differs between species, such that the duplicated *ndhH* coding region ranges in size from a region specifying 32 amino acids in wheat, to 69 in barley, with the *Lolium-Festuca* species possessing 60−67 amino acids.

The phylogenomic dendrogram generated using whole plastome sequence is congruent with other published studies that are based on sequences of the *trn*L-*trn*F spacer region, and the *mat*K gene (Catalán *et al.* 2004; Hand *et al.* 2010; Inda *et al.* 2008). However, the level of divergence between the tall fescue plastome and those of other *Lolium-Festuca* species as observed through the VISTA plot was an unexpected result. Studies of phylogeny based on DNA sequence, molecular genetic marker polymorphism, and morphological variation have consistently positioned tall fescue within the *Schedonorus* subgenus of *Festuca*, and hence more closely related to meadow fescue and the *Lolium* species, than to *F. altissima* and *F. ovina* (Charmet *et al.* 1997; Clayton and Renvoize 1986; Torrecilla and Catalán 2002; Xu and Sleper 1994). It is possible that this incongruence is related to the quality of the tall fescue plastome sequence, a proposition further supported by observation of many single nucleotide indels within the *rbcL*–*psaI* region, compared with other Poaceae species. At the time of publication of the tall fescue plastome sequence, the magnitude of divergence was not obvious, but the comparisons made in the present study reveal clear inconsistencies. To determine whether the unexpected sequence dissimilarity is a byproduct of the sequencing strategy or a genuine evolutionary anomaly, additional plastome sequences should be generated from multiple tall fescue individuals. Given the relative ease with which plastomes can now be sequenced and assembled, as demonstrated here, such resequencing studies are a highly feasible future objective.

### Comparative plastome analysis within the Poaceae family

Plastomes of the *Lolium-Festuca* species possess known grass-specific structural alterations in that each contains the sequence insertion within *rpoC2* but has lost the ORF of *ycf2* within the IR, and an intron within *rpoC1*. Each of these features appears to be common throughout the Poaceae, although the plastome of the grass species *Anomochloa marantoidea* Brogn. still contains the *rpoC1* intron (Morris and Duvall 2010), suggesting that loss of this intron is a more recent evolutionary event. The region of the plastome between *rbcL* and *psaI* has been identified as a “mutational hot-spot” and displays a greater level of interspecies variation than the other grass-specific alterations. One particular feature within this region is the presence of a pseudogene resembling *rpl23*, which has presumably originated after a translocation event, involving insertion of part of the IR into this region of the LSC (Shimada and Sugiura 1989). Studies focused on the presence of this pseudogene have previously indicated variability within the *Lolium* and *Festuca* genera. The *rpl23*′ pseudogene has been reported to be present in tall fescue and *F. ovina* but absent in the plastomes of perennial ryegrass and *F. rubra* (Katayama and Ogihara 1996). This result was obtained using Southern hybridization and was also subsequently achieved through BLAST analysis of the sugarcane *rpl23*′ sequence to the perennial ryegrass and tall fescue plastome sequences (Morris and Duvall 2010). The generation of complete plastome sequences in the present study, however, has revealed two deletions common to all included *Lolium-Festuca* species compared with other Poaceae species, which have effectively removed the majority of the *rpl23′* pseudogene. Based on the available data, it is possible to propose that these deletions occurred at a point predating the origin of the *Festuca* genus, and hence no *Lolium-Festuca* species are likely to contain *rpl23′*. Conflicting previous results with respect to presence of the *rpl23′* pseudogene in *Festuca* and *Lolium* species are therefore possibly due to an inadequate level of resolution associated with the Southern hybridization analyses, due to positive signals arising from residual homology with partial remnants of *rpl23′* for both tall fescue and *F. ovina*.

Similarly, a more ancient deletion event that is possibly common to the entire Pooideae subfamily has occurred within the region in which *accD* pseudogenes have been predicted. The timing of this 173-bp deletion event is more difficult to determine, as it is nested within much larger deleted regions of both the wheat and *B. distachyon* plastomes. A more recent deletion has occurred within *F. ovina*, or an ancestor of this taxon, which has removed an even larger component of the *accD* pseudogene.

Alignment of the *rbcL*–*psaI* region for selected Poaceae family taxa has revealed a number of other deletions that appear to be common to particular subfamilies (such as the c. 1-kb deletion within Panicoideae species maize and sorghum) and others that are diagnostic for either whole tribes, genera or individual species. To further understand the evolutionary timing of these mutational events, comparison of a larger number of species is required. An apparent prediction of this analysis, however, is that the variable presence of the *rpl23′* and *accD* pseudogenes is the product of a number of independent mutation events. Consequently, the sole use of the presence of these genes as a phylogenetic feature will not necessarily generate genuine species relationships, and should therefore be avoided.

### Future applications

Apart from the obvious significance for genome evolutionary and phylogenetic studies, the complete plastome sequences generated in this study are anticipated to be valuable in the field of plant genetic modification. Generation of transformed plants through chloroplast-directed genetic engineering may provide benefits that include high levels of transgene expression, an apparent lack of associated gene silencing, and transgene containment due to restriction to the maternal line (and hence absence of pollen-mediated dispersal) (Han *et al.* 2008). Consequently, plastome-mediated transformation has been successfully used to improve resistance or tolerance to herbicides, insect predation, disease, drought and salt stress (Degray *et al.* 2001; Kota *et al.* 1999; Lee *et al.* 2003; Morris and Duvall 2010; Ruhlman *et al.* 2010; Tu *et al.* 2010). Within the *Lolium-Festuca* complex, transgenic approaches have been implemented in the agriculturally important species perennial ryegrass, Italian ryegrass, tall fescue and meadow fescue, with the aim of improving traits such as abiotic stress tolerance (Cao *et al.* 2009; Han *et al.* 2008), digestibility (Chen *et al.* 2004; Tu *et al.* 2010), resistance to fungal diseases (Dong *et al.* 2007; Takahashi *et al.* 2005) and allergenicity (Bhalla *et al.* 2001; Petrovska *et al.* 2005). Although the transformation approaches used for *Lolium-Festuca* species have not yet involved plastid-directed modification engineering, the availability of plastome sequences from each of these species, as described in this study, now allows for this to be a future possibility.

Ideally, development of transformation techniques using plastome-directed vectors will use species-specific plastome sequence data, rather than relying upon heterologous information. A number of studies have correlated reduced transformation efficiency with decreased sequence identity between flanking sequence within the transformation vector, and the target plastome (Degray *et al.* 2001; Nguyen *et al.* 2005; Ruhlman *et al.* 2010). However, variation between target plastome sites makes it difficult to identify a universal integration site. This effect has been demonstrated for the grass family, such that identical intergenic regions failed to be identified between rice, wheat, barley, and creeping bentgrass despite their close evolutionary affinities (Saski *et al.* 2007). Consequently, efficient plastid transformation is dependent on parallel efforts in both transformation techniques and plastome sequencing (Clarke *et al.* 2011). To date, the most widely used site within the plastome for transgene integration has been the transcriptionally active *trn*I-A intergenic region within the IR, resulting in the highest reported levels of expression (De Cosa *et al.* 2001; Verma and Daniell 2007). This intergenic region is identical among the six *Lolium-Festuca* species analyzed in this study and so may prove highly effective for chloroplast-mediated transformation of *Lolium-Festuca* grasses.

## Supplementary Material

Supporting Information
