# Supplementary material for: Plastome Sequence Determination and Comparative Analysis for Members of the Lolium-Festuca Grass Species Complex
Source: G3 (Bethesda). 2013 Apr 1;3(4):607–16. doi: 10.1534/g3.112.005264 (PMC3618348; doi:10.1534/g3.112.005264)
Supplement: Supporting Information [file supp_3_4_607__index.html]

Plastome Sequence Determination and Comparative Analysis for Members of the Lolium-Festuca Grass Species Complex — Supporting Information 

# Plastome Sequence Determination and Comparative Analysis for Members of the *Lolium*-*Festuca* Grass Species Complex

## Supporting Information for Hand *et al.*, 2013

**Files in this Data Supplement:**

- Table S1 - Details of the reference genomes used to predict the level of plastid or bacterial genome sequence present in the total sequencing output of each species (PDF, 126 KB)
