# Supplementary material for: Plastome Sequence Determination and Comparative Analysis for Members of the Lolium-Festuca Grass Species Complex
Source: G3 (Bethesda). 2013 Apr 1;3(4):607–16. doi: 10.1534/g3.112.005264 (PMC3618348; doi:10.1534/g3.112.005264)
Supplement: Supporting Information [file supp_g3.112.005264_TableS1.pdf]

G3 · Genes | Genomes | Genetics

Plastome sequence determination and comparative analysis for members of the *Lolium-Festuca* grass species complex

Melanie L. Hand[*,†,‡], German C. Spangenberg[*,†,‡], John W. Forster[*,†,‡], Noel O.I. Cogan[*,†]

[*]Department of Primary Industries, Biosciences Research Division, AgriBio, the Centre for AgriBioscience, La Trobe University Research and Development Park, Bundoora, Victoria 3083, Australia

[†]Dairy Futures Cooperative Research Centre, Australia

[‡]La Trobe University, Bundoora, Victoria 3086, Australia

DOI: 10.1534/g3.112.005264

Table S1 Details of the reference genomes used to predict the level of plastid or bacterial genome sequence present in the total sequencing output of each species.

Source	Species and genome description
NC_009950.1	*Lolium perenne* chloroplast, complete genome
FJ466687.2	*Festuca arundinacea* cultivar KY-31 chloroplast, complete genome
EF115541.1	*Hordeum vulgare* subsp. *vulgare* cultivar Morex chloroplast, complete genome
NC_002762.1	*Triticum aestivum* chloroplast, complete genome
EU325680.1	*Brachypodium distachyon* cultivar Bd21 chloroplast, complete genome
NC_000908.2	*Mycoplasma genitalium* G37, complete genome
NC_002696.2	*Caulobacter crescentus* CB15 chromosome, complete genome
NC_003919.1	*Xanthomonas axonopodis* pv. *citri* str. 306 chromosome, complete genome
NC_004923.1	*Aeromonas salmonicida salmonicida* A449 plasmid pAsa1, complete sequence
NC_004924.1	*Aeromonas salmonicida salmonicida* A449 plasmid pAsa3, complete sequence
NC_004925.1	*Aeromonas salmonicida salmonicida* A449 plasmid pAsa2, complete sequence
NC_006270.3	*Bacillus licheniformis* ATCC 14580 chromosome, complete genome
NC_006582.1	*Bacillus clausii* KSM-K16, complete genome
NC_007488.1	*Rhodobacter sphaeroides* 2.4.1 plasmid B, complete sequence
NC_007489.1	*Rhodobacter sphaeroides* 2.4.1 plasmid C, complete sequence
NC_007490.1	*Rhodobacter sphaeroides* 2.4.1 plasmid D, complete sequence
NC_007493.1	*Rhodobacter sphaeroides* 2.4.1 chromosome 1, complete sequence
NC_007494.1	*Rhodobacter sphaeroides* 2.4.1 chromosome 2, complete sequence
NC_007614.1	*Nitrosospira multiformis* ATCC 25196 chromosome, complete genome
NC_008009.1	*Candidatus Koribacter* versatilis Ellin345 chromosome, complete genome
NC_008255.1	*Cytophaga hutchinsonii* ATCC 33406 chromosome, complete genome
NC_008686.1	*Paracoccus denitrificans* PD1222 chromosome 1, complete sequence
NC_008687.1	*Paracoccus denitrificans* PD1222 chromosome 2, complete sequence

Source	Species and genome description
NC_008688.1	*Paracoccus denitrificans* PD1222 plasmid 1, complete sequence
NC_008765.1	*Acidovorax* sp. JS42 plasmid pAOVO01, complete sequence
NC_008766.1	*Acidovorax* sp. JS42 plasmid pAOVO02, complete sequence
NC_008782.1	*Acidovorax* sp. JS42 chromosome, complete genome
NC_008825.1	*Methylibium petroleiphilum* PM1 chromosome, complete genome
NC_008826.1	*Methylibium petroleiphilum* PM1 plasmid RPME01, complete sequence
NC_009007.1	*Rhodobacter sphaeroides* 2.4.1 plasmid A, partial sequence
NC_009008.1	*Rhodobacter sphaeroides* 2.4.1 plasmid E, complete sequence
NC_009138.1	*Herminiimonas arsenicoxydans* chromosome, complete genome
NC_009348.1	*Aeromonas salmonicida* subsp. *salmonicida* A449, complete genome
NC_009349.1	*Aeromonas salmonicida* subsp. *salmonicida* A449 plasmid 4, complete sequence
NC_009350.1	*Aeromonas salmonicida* subsp. *salmonicida* A449 plasmid 5, complete sequence
NC_009441.1	*Flavobacterium johnsoniae* UW101 chromosome, complete genome
NC_009483.1	*Geobacter uraniireducens* Rf4 chromosome, complete genome
NC_009613.1	*Flavobacterium psychrophilum* JIP02/86 chromosome, complete genome
NC_009617.1	*Clostridium beijerinckii* NCIMB 8052 chromosome, complete genome
NC_010162.1	*Sorangium cellulosum* 'So ce 56' chromosome, complete genome
NC_010394.1	*Mycobacterium abscessus* ATCC 19977 unnamed plasmid, complete sequence
NC_010397.1	*Mycobacterium abscessus* ATCC 19977 chromosome 1, complete sequence
NC_010524.1	*Leptothrix cholodnii* SP-6 chromosome, complete genome
NC_010571.1	*Opitutus terrae* PB90-1 chromosome, complete genome
NC_010655.1	*Akkermansia muciniphila* ATCC BAA-835 chromosome, complete genome
NC_010794.1	*Methylacidiphilum infernorum* V4, complete genome
NC_010830.1	*Candidatus Amoebophilus asiaticus* 5a2 chromosome, complete genome
NC_010943.1	*Stenotrophomonas maltophilia* K279a chromosome, complete genome

Source	Species and genome description
NC_011297.1	*Dictyoglomus thermophilum* H-6-12, complete genome
NC_011880.1	*Cyanothece* sp. PCC 7425 plasmid pP742501, complete sequence
NC_011882.1	*Cyanothece* sp. PCC 7425 plasmid pP742503, complete sequence
NC_011884.1	*Cyanothece* sp. PCC 7425 chromosome, complete genome
NC_011885.1	*Cyanothece* sp. PCC 7425 plasmid pP742502, complete sequence
NC_012791.1	*Variovorax paradoxus* S110 chromosome 1, complete sequence
NC_012792.1	*Variovorax paradoxus* S110 chromosome 2, complete genome
NC_013037.1	*Dyadobacter fermentans* DSM 18053 chromosome, complete genome
NC_013061.1	*Pedobacter heparinus* DSM 2366 chromosome, complete genome
NC_013124.1	*Acidimicrobium ferrooxidans* DSM 10331 chromosome, complete genome
NC_013132.1	*Chitinophaga pinensis* DSM 2588 chromosome, complete genome
NC_013173.1	*Desulfomicrobium baculatum* DSM 4028, complete genome
NC_013190.1	*Candidatus Accumulibacter phosphatis* clade IIA str. UW-1 plasmid pAph02, complete sequence
NC_013191.1	*Candidatus Accumulibacter phosphatis* clade IIA str. UW-1 plasmid pAph03, complete sequence
NC_013193.1	*Candidatus Accumulibacter phosphatis* clade IIA str. UW-1 plasmid pAph01, complete sequence
NC_013194.1	*Candidatus Accumulibacter phosphatis* clade IIA str. UW-1 chromosome, complete genome
NC_013730.1	*Spirosoma linguale* DSM 74 chromosome, complete genome
NC_013731.1	*Spirosoma linguale* DSM 74 plasmid pSLIN01, complete sequence
NC_014148.1	*Planctomyces limnophilus* DSM 3776 chromosome, complete genome
NC_014149.1	*Planctomyces limnophilus* DSM 3776 plasmid pPLIM01, complete sequence
NC_014153.1	*Thiomonas intermedia* K12 chromosome, complete genome

**Source**	**Species and genome description**
NC_014501.1	*Cyanothece* sp. PCC 7822 chromosome, complete genome
NC_014502.1	*Cyanothece* sp. PCC 7822 plasmid Cy782203, complete sequence
NC_014503.1	*Cyanothece* sp. PCC 7822 plasmid Cy782204, complete sequence
NC_014504.1	*Cyanothece* sp. PCC 7822 plasmid Cy782205, complete sequence
NC_014533.1	*Cyanothece* sp. PCC 7822 plasmid Cy782201, complete sequence
NC_014534.1	*Cyanothece* sp. PCC 7822 plasmid Cy782202, complete sequence
NC_014535.1	*Cyanothece* sp. PCC 7822 plasmid Cy782206, complete sequence
NC_014655.1	*Leadbetterella byssophila* DSM 17132 chromosome, complete genome
NC_014738.1	*Riemerella anatipestifer* DSM 15868 chromosome, complete genome
NC_014750.1	*Marivirga tractuosa* DSM 4126 plasmid pFTRAC01, complete sequence
NC_014759.1	*Marivirga tractuosa* DSM 4126 chromosome, complete genome
NC_014816.1	*Asticcacaulis excentricus* CB 48 chromosome 1, complete sequence
NC_014817.1	*Asticcacaulis excentricus* CB 48 chromosome 2, complete sequence
NC_014818.1	*Asticcacaulis excentricus* CB 48 plasmid pASTEX01, complete sequence
NC_014819.1	*Asticcacaulis excentricus* CB 48 plasmid pASTEX02, complete sequence
NC_014960.1	*Anaerolinea thermophila* UNI-1, complete genome
